# Lumbar Disc Herniation Automatic Detection in Magnetic Resonance Imaging Based on Deep Learning

**DOI:** 10.3389/fbioe.2021.708137

**Published:** 2021-08-19

**Authors:** Jen-Yung Tsai, Isabella Yu-Ju Hung, Yue Leon Guo, Yih-Kuen Jan, Chih-Yang Lin, Tiffany Ting-Fang Shih, Bang-Bin Chen, Chi-Wen Lung

**Affiliations:** ^1^ Department of Digital Media Design, Asia University, Taichung, Taiwan; ^2^ Department of Nursing, Chung Hwa University of Medical Technology, Tainan, Taiwan; ^3^ Environmental and Occupational Medicine, College of Medicine, National Taiwan University (NTU) and NTU Hospital, Taipei, Taiwan; ^4^ Graduate Institute of Environmental and Occupational Health Sciences, College of Public Health, National Taiwan University, Taipei, Taiwan; ^5^ National Institute of Environmental Health Sciences, National Health Research Institutes, Miaoli, Taiwan; ^6^ Rehabilitation Engineering Lab, Department of Kinesiology and Community Health, University of Illinois at Urbana-Champaign, Champaign, IL, United States; ^7^ Department of Electrical Engineering, Yuan Ze University, Chung-Li, Taiwan; ^8^ Department of Medical Imaging and Radiology, National Taiwan University (NTU) Hospital and NTU College of Medicine, Taipei, Taiwan; ^9^ Department of Creative Product Design, Asia University, Taichung, Taiwan

**Keywords:** object detection, medical image, YOLO, low back pain, data augmentation

## Abstract

**Background:** Lumbar disc herniation (LDH) is among the most common causes of lower back pain and sciatica. The causes of LDH have not been fully elucidated but most likely involve a complex combination of mechanical and biological processes. Magnetic resonance imaging (MRI) is a tool most frequently used for LDH because it can show abnormal soft tissue areas around the spine. Deep learning models may be trained to recognize images with high speed and accuracy to diagnose LDH. Although the deep learning model requires huge numbers of image datasets to train and establish the best model, this study processed enhanced medical image features for training the small-scale deep learning dataset.

**Methods:** We propose automatic detection to assist the initial LDH exam for lower back pain. The subjects were between 20 and 65 years old with at least 6 months of work experience. The deep learning method employed the YOLOv3 model to train and detect small object changes such as LDH on MRI. The dataset images were processed and combined with labeling and annotation from the radiologist’s diagnosis record.

**Results:** Our method proves the possibility of using deep learning with a small-scale dataset with limited medical images. The highest mean average precision (mAP) was 92.4% at 550 images with data augmentation (550-aug), and the YOLOv3 LDH training was 100% with the best average precision at 550-aug among all datasets. This study used data augmentation to prevent under- or overfitting in an object detection model that was trained with the small-scale dataset.

**Conclusions:** The data augmentation technique plays a crucial role in YOLOv3 training and detection results. This method displays a high possibility for rapid initial tests and auto-detection for a limited clinical dataset.

## Highlight

1. Auto-detect the location of lumbar disc herniation for MRI images based on deep learning.
2. The MRI images with data augmentation were successful in training small-scale deep learning datasets.
3. It provided a solution for medical images with limited cases for data imbalance.
4. It showed the possibility for initial rapid tests in real time and auto-detection for clinical diagnosis.

## Introduction

The lumbar disc herniation (LDH) is caused by complex situations. Excessive pressure on the spinal column squeezes the intervertebral discs to present lower back pain, sciatica, or radiating pain symptoms (Amin, Andrade, and Neuman, 2017; Scheer et al., 1996). Some reports pointed out that 70–80% of adults have experienced lower back pain (Paolucci et al., 2019). People experience lower back discomfort, sudden pain, or continuous stabbing sensation, and sometimes even more serious LDH symptoms that affect their daily activities and working performance. The medical expenses for lumbar conditions such as medicines and surgery have increased by 177% from 2004 to 2015 (Martin et al., 2019). These personal expenditures cause a great burden on social welfare with its already limited financial resources.

As if this high prevalence was not enough, the number of pathological findings might be even higher. A study shows 98 asymptomatic subjects were examined using magnetic resonance imaging (MRI). In magnetic resonance imaging, many of these asymptomatic subjects presented abnormal discs, 52% of people presented bulging discs, and 38% of people presented more than one abnormal intervertebral disk (Jensen et al., 1994). Another study showed that MRI scans display abnormal images for lumbar disc herniation in 90% of asymptomatic people at the L4-L5 and L5-S1 vertebrae (Faur, Patrascu, Haragus, and Anglitoiu, 2019).

Medical personnel and doctors require long and intensive training to interpret and analyze biomedical images. The well-trained radiologist would be able to examine MRIs with the naked eye and classify the signs of LDH to bulging, protrusion, extrusion, and sequestration. Due to the high workload, doctors can be affected by stress and fatigue from interpreting complex biomedical images. Some reports indicate the advantage of the existing computing assistant medical diagnosis and treatment from the artificial neural network (ANN) to deep learning (Azimi et al., 2020). The advance of computing procession could deal with huge data, and complex calculated facts. Although, it still requires professional adjustment and more advanced hardware to support.

Deep learning models are trained to recognize images with high speed and accuracy. Deep learning demonstrates precision technology to provide better medical quality in a clinical setting. This could increase the speed of processing and diagnosis as well as assisting medical personnel and doctors in finding unnoticed lesions. The deep learning method is applied to high-volume and repeatable processes to recognize biomedical images. Deep learning methods use advanced graphic processing units (GPUs) to calculate the featured figures and auto-classify or identify the image’s objects (Tsai et al., 2020). At the moment, there is a shortage of confirmed diagnosis MRI images for the lumbar vertebrae dataset. Therefore, it is essential to conduct a deep learning model and enlarge the lumbar MRI data collection (Zhou, Liu, Chen, Gu, and Sui, 2019).

In addition to image recognition, deep learning is tasked to quickly or repeatedly find significant objects in the biomedical image, such as detecting abnormal and suspicious lesions in the clinical diagnosis process. RCNN (Region-based Convolutional Neural Network) provides new methods to detect objects in images. The RCNN algorithm uses a marked object region proposal method to train CNN and then classify objects. Although the later version is intended to improve the processing speed as Fast-RCNN and Faster-RCNN, these complex object detection methods are still not efficient deep learning models.

Other studies employed YOU ONLY LOOK ONCE (YOLO) to train biomedical image detection and prediction in real time, using the anchor base and intersection over union deep learning technique (Ozturk et al., 2020; Safdar, Alkobaisi, and Zahra, 2020; Ünver & Ayan, 2019). The YOLO algorithm utilizes a grid to anchor the training target and detect and predict the object images. Varçın et al. used the YOLOv3 algorithm combined with the MobileNet deep learning model to detect lumbar spondylolisthesis signs and symptoms (Varçın, Erbay, Çetin, Çetin, and Kültür, 2021). Ozturk et al. proposed the YOLO predecessor architecture, DarkNet-19 classifier to focus on small parts detection on the deeper convolutional layer (Ozturk et al., 2020).

The deep learning model requires huge numbers of image datasets to train and establish the best model. In addition to designing different deep learning architectures, researchers also pursue better training results. Large numbers of diagnosed images with pathologies are difficult to obtain and access for deep learning dataset training. This is a serious limitation for biomedical image researchers as collecting and storing images from a multitude of variable conditions and formats is tedious.

Data augmentation is therefore an important process for increasing the data scale and reducing the data imbalance error. The basic data augmentation techniques use biomedical image photometric and geometric transformation (Hussain et al., 2017; Perez, Vasconcelos, Avila, and Valle, 2018). Some studies employ self-designed convolutional layer architecture to recognize biomedical images in X-rays, computed tomography, and magnetic resonance imaging (Cai et al., 2016; Forsberg, Sjöblom, and Sunshine, 2017; Zhou et al., 2019). These studies show the potential of new models for constructing different convolutional neural networks to train in deep learning. Zhou et al. proposed contrasting images compared to the kernel to the framework’s convolutional layer region (Zhou et al., 2019). Forsberg et al. showed the construction of two pipelines to detect and label within two convolutional neural networks (CNNs). The target is then trained and the detected images are output (Forsberg et al., 2017). They intended to provide different labeling mechanisms in biomedical image recognition.

This study processes the enhanced medical image features for training the small-scale deep learning dataset. We propose automatic detection to assist with the initial lumbar vertebrae exam for lower back pain. Our dataset presents the relatively small abnormal lumbar intervertebral disc features distributed in many locations. The object detection method uses a boundary box to detect abnormal lumbar intervertebral disc parts in the region of the first lumbar vertebra (L1), second lumbar vertebra (L2), third lumbar vertebra (L3), fourth lumbar vertebra (L4), fifth lumbar vertebra (L5), and first sacral vertebra (S1).

## Methods

### Subject and Data Collection

This was a retrospective study. The subjects were recruited from wholesale market workers and walk-in clinic patients who sought treatment in the Internal Medicine clinic and were diagnosed with upper respiratory infections (URIs). They were invited to participate in a study regarding spine and bone health from 2009 to 2011. Before participating in the study, all participants received written and oral information regarding the study procedures and potential adverse effects and signed informed consent. The inclusion and exclusion criteria of the study were described in detail in another study (Y. J. Hung et al., 2014). The protocol and consent forms of the study were reviewed and approved by the National Taiwan University Hospital Research Ethics Committee. We reviewed the MRI scans of 168 male participants from the examined data. The age ranged from 20 to 65 years. Some subjects had a medical history of lower back pain. The doctor may advise them for further diagnosis and treatment after the MRI examination (I. Y. Hung, Shih, Chen, and Guo, 2021).

All participants received a lumbar vertebrae scan using a GE 1.5-T unit (General Electric Medical Systems, Milwaukee, Wisconsin) with a spine array coil (12.7 × 27.9 cm) at National Taiwan University Hospital. The lumbar MRI images were stored as DICOM files (Digital Imaging and Communications in Medicine). The system set these T2-weighted images to 4 mm slice thickness, and the sagittal and axial field of view was 28 by 20 cm. Radiologist members of this research team interpreted these lumbar MRI images and recorded the process during the standard diagnostic procedure. The radiological diagnostic criteria for LDH were signs of bulging, protrusion, extrusion, and sequestration on MRI. They ignored the participants’ occupational status and medical history for a blind examination in this evaluation process (I. Y. Hung et al., 2021).

### Architecture of Deep Learning

This study employed the YOLOv3 model to train and detect small object changes such as LDH on MRI. YOLO’s first algorithm used grouped grids in the image as the bounding box to improve the speed and presented great real-time object detection (Redmon, Divvala, Girshick, and Farhadi, 2016). The earlier version, YOLOv2, used the anchor boxes and multiscale training to improve the prediction accuracy. YOLOv3 improved previous algorithms to deal with higher resolution images and detect small objects. YOLOv3’s report indicated it was capable of performing at detecting objects in 2018.

**FIGURE 1:**
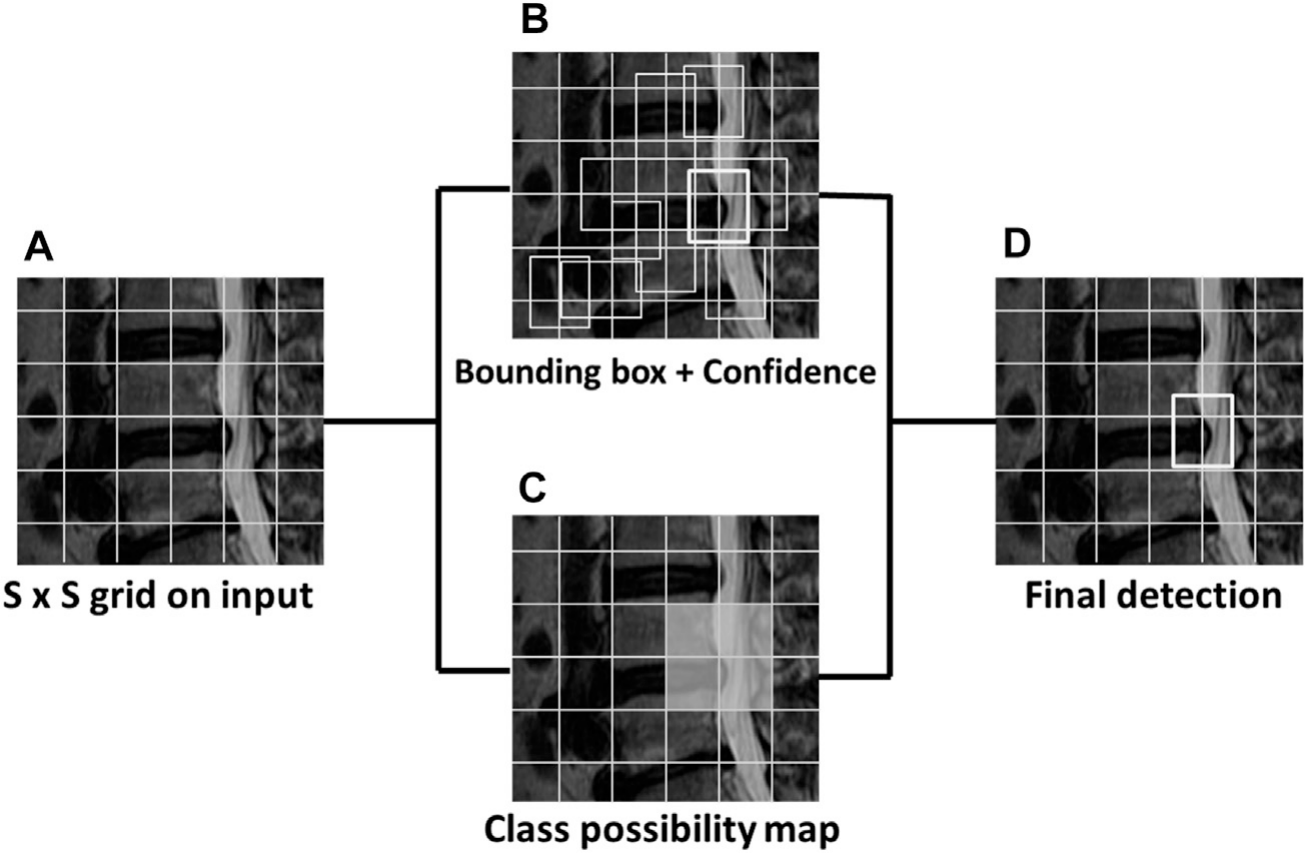
The sample MRI of YOLOv3 detects lumbar disc herniation (LDH). The first image **(A)** is divided into S x S grids. The bounding box and class possibility then calculate **(B) (C)** into the confidence score to output the predicted image **(D)** with the bounding box.

YOLOv3 proposes multiscale prediction with independent logistic classifiers to replace the Softmax function. In the multiscale prediction process, the initial image was divided into an S x S grid cell. It then checks the center of each located object in each grid area and refers to each bounding box as “B.” “C” represents the number of predicted classes. The bounding box and class possibility calculate the confidence score and then output the box’s predicted image (Eq. 1). The object detection was calculated using the four bounding box offsets and one confidence score with the image grid. This was similar to the feature pyramid networks to extract the image features. The prediction generates the final output of the tensor with (S x S) *B * (1 + 4 + C) (Ünver and Ayan, 2019) (Figure 1).

Confidence =Pr(Object) ∗ IoU(pred, truth). (1)

**FIGURE 2:**
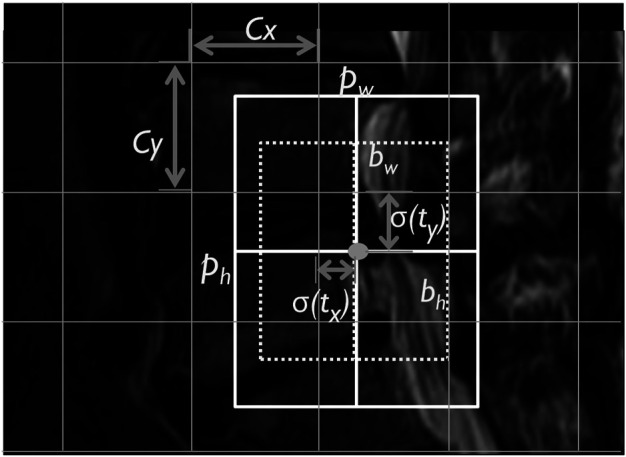
The lumbar MRI bounding box YOLOv3 predicted box image. *Note*. C_x_, C_y_, corner: p_w_, p_h_, bounding box width and height; b_w_, b_h_, predicted width and height; and $\sigma \left(t_{x}\right)$, $\sigma \left(t_{y}\right)$, sigmoid function.

YOLOv3 architecture predicts the LDH location with the bounding box in MRI. It uses four coordinates of width, height, offset point x, and y of the bounding box (Figure 2) (Redmon and Farhadi, 2018). The offset coordinates t_x_, t_y_, t_w_, t_h_ calculate an object classification within the bounding box. YOLOv3 uses logistic regression to predict the object. It takes the largest bounding box overlap threshold and ground truth and ignores the others. YOLOv3 also uses the binary cross-entropy loss function to replace the classification loss’s mean squared error to predict the object class with object confidence. It uses bounding boxes to denote the object as binary classification predicts.

The YOLOv3 framework builds the convolutional neural network called DarkNet-53, using the feature extractor with 53 layers and a shortcut route (Table 1). The network layer increases deeper than 50 layers to solve the learning gradient problem in the neural network model. Except for the convolution layers deeper than YOLOv2 Darknet-19, YOLOv3 presents a better prediction for small object performance through the multiscale detector with DarkNet-53.

**TABLE 1:** Lumbar MRI DarkNet-53 architecture.

	Type	Filters	Size	Output
	Convolutional	32	3 × 3	512 × 512
	Convolutional	64	3 × 3/2	256 × 256
	Convolutional	32	1 × 1	–
1X	Convolutional	64	3 × 3	–
	Shortcut layer	256 × 256		
	Convolutional	128	3 × 3/2	128 × 128
	Convolutional	64	1 × 1	V
2X	Convolutional	128	3 × 3	–
	Shortcut layer	128 × 128		
	Convolutional	256	3 × 3/2	64 × 64
	Convolutional	128	1 × 1	–
8X	Convolutional	256	3 × 3	–
	Shortcut layer	64 × 64		
	Convolutional	256	3 × 3/2	32 × 32
	Convolutional	128	1 × 1	–
8X	Convolutional	256	3 × 3	–
	Shortcut layer	32 × 32		
	Convolutional	256	3 × 3/2	16 × 16
	Convolutional	128	1 × 1/1	–
4X	Convolutional	256	3 × 3/1	–
	Shortcut layer	16 × 16		
	Avgpool Connected Softmax	–	Global 1000	–

### Dataset

**FIGURE 3:**
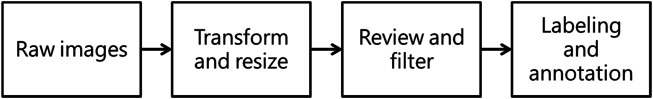
Data normalization process flow for object detection with deep learning.

Before training the deep learning model, all images must be normalized, reviewed, and turned into organized data (Zhao, Zheng, Xu, and Wu, 2019) (Figure 3). This study uses T2-weighted images for obtaining better darkness and brightness features from the DICOM raw data. The DICOM images were reformatted into JPEG images and resized to a resolution of 512 × 512. These JPG images contain 11 sliced images of the lumbar vertebrae MRI in sagittal view. Each of the LDH is comprised of five MRI images symmetrical from the middle to both sides (Forsberg et al., 2017).

**FIGURE 4:**
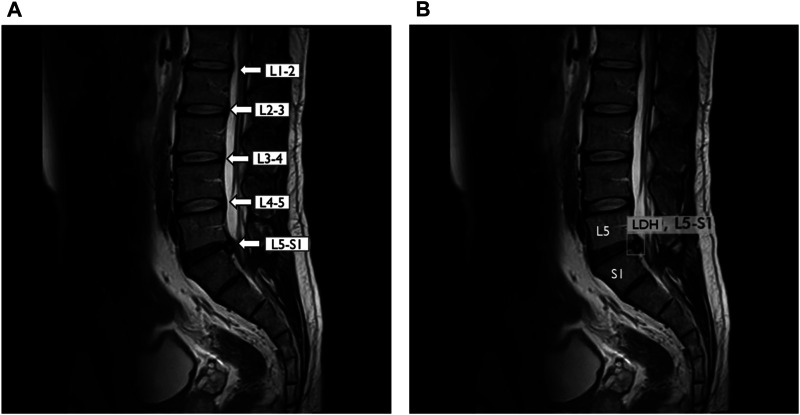
YOLOv3 detected lumbar disc herniation (LDH) location with MRI at L5-S1. **(A)** Lumbar MRI show clearly each lumbar disc from L1 to S1. **(B)** Same lumbar MRI display LDH boundary box at L5–S1 by YOLOv3. L1, first lumbar vertebra; L2, second lumbar vertebra; L3, third lumbar vertebra; L4, fourth lumbar vertebra; L5, fifth lumbar vertebra; S1, first sacral vertebra.

This study included 714 raw images. Then, these images were divided into three groups consisting of 77, 20, and 3% of the images for training, validation, and test of LDH MRI images. For processing by the deep learning algorithm, we made sets consisting of 50, 150, 350, and 550 MRI images. This study further processed the training and validation dataset to combine with the labeling and annotation from the radiologist’s diagnosis record. The lumbar vertebrae and disc section label the MRI images from L1-L2, L2-L3, L3-L4, L4-L5, and L5-S1. YOLOv3 was trained and predicts small object location such as LDH on MRI (Figure 4). The PC hardware settings that run YOLOv3 consist of a CPU Intel Core i7-8700, 16G RAM, and NVIDIA Geforce RTX 2080 Super GPUs with Windows 10, 64 bits.

### Data Augmentation

Many factors affect deep learning, including an insufficient number of images in the dataset, leading to overfitting or underfitting. The earlier studies use data augmentation to prevent data imbalance and reduce overfitting for deep learning (Abdelhafiz, Yang, Ammar, & Nabavi, 2019). The typical data augmentation types for deep learning include Flips, Gaussian Noise, Jittering, Scaling, Powers, Gaussian Blur, Rotations, and Shears (Hussain et al., 2017).

The augmentation changes image pixels and geometry using blur, rotation, and random crop to increase the image feature for image recognition (Dao et al., 2019). The skill of augmentation changes image pixels, shape, and geometry such as image flipping, rotation, scales size, skew, Gaussian noise, and blur for increasing the image feature to training. Hussain et al. (2017) indicated that image flipping, commonly known as mirroring did not increase distinguishable features in deep learning (Z. Hussain et al., 2017). Both training and validation accuracies were higher than 88% when using the image features augmented by rotation (Z. Hussain et al., 2017). Another report proposed to change image pixel brightness and contrast to improve the deep learning training results (Sánchez-Peralta, Picón, Sánchez-Margallo, and Pagador, 2020). There are many methods of data augmentation. However, for leading to better results, we selected the following three types: rotation, brightness, and contrast.

**FIGURE 5:**
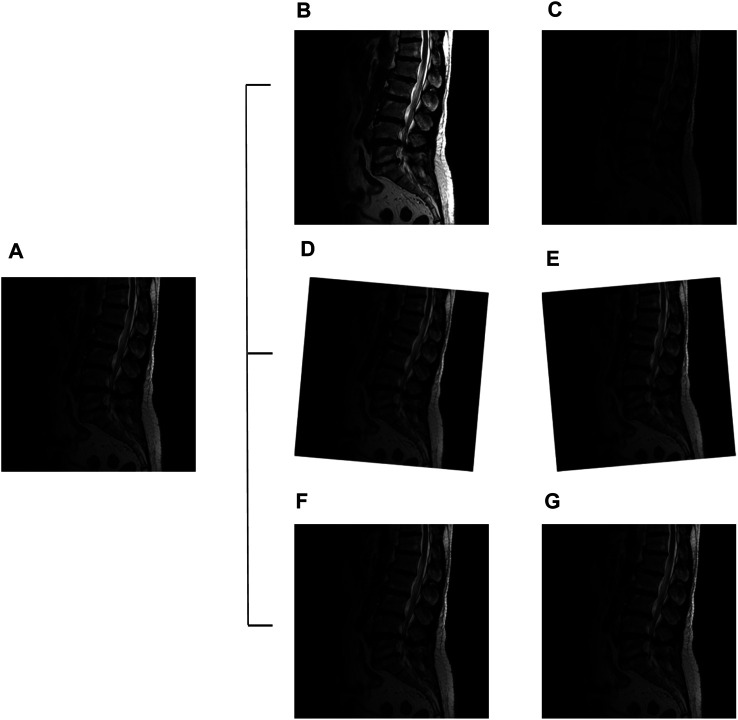
Lumbar images augmentation: original image **(A)**, exposure + 2.1 **(B)**, exposure −2.1 **(C)**, angle +5° **(D)**, angle −5° **(E)**, contrast +1.5 **(F)**, and contrast −1.5 **(G)**.

The image augmentation improves the deep learning model training results (Dao et al., 2019; Z.; Hussain et al., 2017). Therefore, this study used MRI image data augmentation such as rotation, contrast, and brightness to simulate real-life situations. We use different strategies employed in these augmentation settings to increase the volume and features of the images. The rotational changes were used to simulate different positions of patients during MRI acquisition. In addition, each LDH image had adjusted contrast and brightness to simulate different magnetic resonance machines. These data augmentation strategies provide the solution to overly homogenous datasets and increase the image quantity for better results. The MRI scanning also occurred in the same setting but generated different contrast results. The YOLOv3 CFG file set the image rotation angle at 10° ( ±5°) and exposure 2.1 (Perez et al., 2018; Sadykova, Pernebayeva, Bagheri, and James, 2019) (Figure 5).

### Evaluation

YOLOv3 algorithm uses the confidence score to predict the object-based bounding box (Eq. 1). It usually uses the average precision (AP) and the mean average precision (mAP) to evaluate the training and validate the YOLO results on the LDH regions from L1-L2, L2-L3, L3-L4, L4-L5, and L5-S1. However, the LDH cases distribute uneven regions to cause deep learning imbalanced data problems. This does not seem suitable for evaluating the model using only AP and mAP. Therefore, the other evaluation index employed the recall rate (Recall), F1 score, Dice coefficient (Dice), and Jaccard index (Jac) to evaluate LDH detection YOLOv3 model training results. The definitions of the Recall, F1 score, Dice, and Jac are as follows:

$$Recall=\frac{TP}{TP+FN},$$ (2)

$$F1=2\frac{precision*recall}{precision+recall},$$ (3)

$$Dice=\frac{2\;x\;TP}{\left(TP+FP\right)+\left(TP+FN\right)},$$ (4)

$$Jac=\frac{TP}{TP+FN+FP}.$$ (5)

Note: TP, true positive; TN, true negative; FP, false positive; FN, false negative; Dice, dice coefficient; and Jac, Jaccard index.

## Results

The YOLOv3 training results displayed the mAP and AP value of each lumbar vertebra region with different amounts of images, datasets including the 50 images with data augmentation (50-aug), 150 images data with augmentation (150-aug), 350 images data with augmentation (350-aug), and 550 images data with augmentation (550-aug). It took 84 h to train each LDH images group. Furthermore, the detection time in each group was less than 1 second for 50-aug and 150-aug, 2 seconds for 350-aug, and 3 seconds for 550-aug. The highest mAP was 92.4% at the 550-aug, and the second-highest mAP was 86.6% at 350-aug. The other group of results in this study displays AP from each lumbar vertebra region (Table 2).

**TABLE 2:** YOLOv3 training results (%) of lumbar disc herniation (LDH) in different locations.

	Dataset	Score	Lumbar disc herniation (LDH) locations				
			L1-L2	L2-L3	L3-L4	L4-L5	L5-S1
AP (%)	50-aug	64.8	23.3	61.9	80.0	86.2	72.5
	150-aug	78.1	77.9	69.9	76.6	87.2	78.7
	350-aug	86.6	68.5	84.7	93.0	91.2	95.6
	550-aug	92.4	100.0	86.2	92.9	88.7	93.9
Precision (%)	50-aug	56.8	13.7	45.1	68.9	79.1	65.3
	150-aug	77.0	36.4	80.6	84.2	78.5	82.3
	350-aug	86.8	66.7	81.3	90.4	88.6	87.7
	550-aug	87.2	69.7	83.0	92.8	87.4	88.0

Note: AP, Average Precision; L1, first lumbar vertebra; L2, second lumbar vertebra; L3, third lumbar vertebra; L4, fourth lumbar vertebra; L5, fifth lumbar vertebra; and S1, first sacral vertebra.

The other dataset groups showed different AP outcomes in each of the LDH regions. In the LDH region L1-L2, the lowest AP was 23.3% in the 50-aug dataset, and the highest AP was 100% in the 550-aug. This region showed extreme differences to all of the dataset groups. The LDH YOLOv3 training displayed the best AP 100% in the 550-aug among all datasets. The second-best result was 95.6%, shown in the 350-aug of LDH group. The AP only falls short of 80% in the 150-aug and 50-aug datasets.

The YOLOv3 evaluation index shows the list of comparisons with different groups of LDH datasets (Table 3). The F1 score was the same at 89% in the 350-aug and 550-aug groups. The highest Jaccard index was 93.0% in the 350-aug group. The recall rate was 92% in the 550-aug group.

**TABLE 3:** Evaluation index of YOLOv3 lumbar disc herniation (LDH) (%).

Dataset	IoU	Precision	Recall	F1	Accuracy	Sensitivity	Jac	Dice
50-aug	42.8	56.7	91.3	70.0	62.3	91.3	53.8	70.0
150-aug	59.1	76.9	80.6	78.7	69.9	80.6	65.0	78.8
350-aug	70.4	86.8	90.8	88.7	81.1	90.8	79.8	88.8
550-aug	73.6	87.2	91.7	89.4	81.1	91.7	80.8	89.4

Note: intersection over union (IoU), recall rate (Recall), Jaccard index (Jac), Dice coefficient (Dice).

## Discussion

This study proves the possibility of using deep learning with a small-scale dataset with limited medical images. We used the image process technique to increase the LDH images deep learning feature using data augmentation. The YOLOv3 detection results were impressive in detecting the LDH region. The results are consistent with the hypothesis of this study.

**FIGURE 6:**
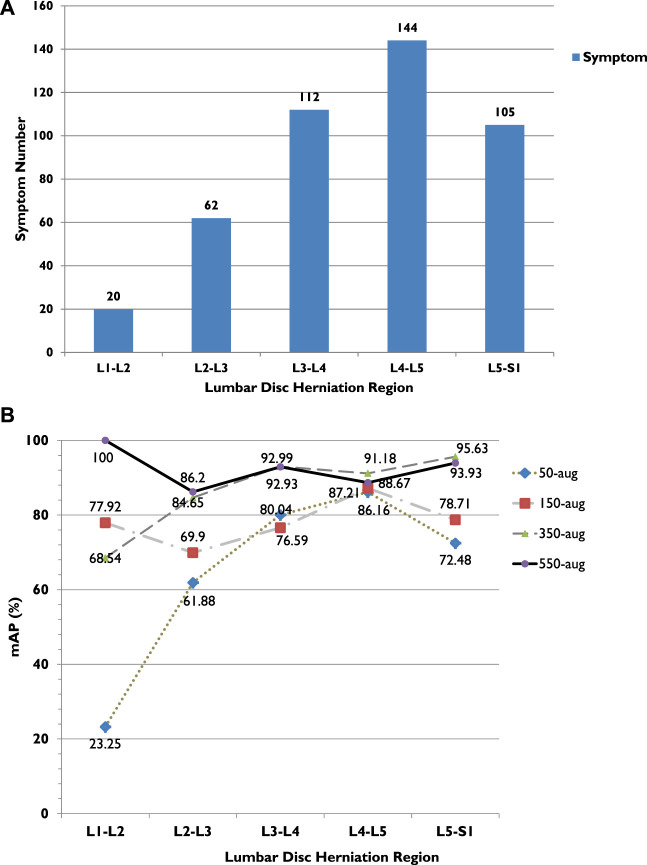
**(A)** LDH cases in different lumbar vertebrae regions. **(B)** LDH cases and average precision (AP) related trend in different lumbar vertebrae regions. L1, first lumbar vertebra; L2, second lumbar vertebra; L3, third lumbar vertebra; L4, fourth lumbar vertebra; L5, fifth lumbar vertebra; S1, first sacral vertebra.

In our method, the model displays the small-scale dataset training performance with the YOLOv3 algorithm. Among the four dataset groups, the highest mAP was 92.4% in the 500-aug group (Table 2). Compared with other datasets, the second-highest mAP was 86.6% in the 350-aug set. The detection results indicate that each mAP value increases with the different dataset changes from 50-aug to 550-aug with the data augmentation technique. In addition to mAP, each lumbar level also shows the precision related to the case quantities in Figure 6A. For example, the lowest precision, 13.7% in 50-aug located at L1-L2 for training with 20 cases and the highest precision of 92.8% in 550-aug at L3-L4 for training with 144 cases.

The Intersection over Union (IoU) defines the value to divide the area of overlap by union with a bounding box and ground truth. To evaluate the YOLOv3 performance, we calculated the detection results with the number of true positives (TP), false positives (FP), true negatives (TN), and false negatives (FN) as the evaluation index metrics (Eq. 2–5). From the precision aspect, both 500-aug and 350-aug groups were the same at 87%, which means the model was trained well to detect LDH in MRI. It shows a recall rate of 92 and 91% in reference to the correct detection.

The highest achieved recall rate in 550-aug was 92%, and both 350-aug and 50-aug obtained the same recall rate of 91%. Although the recall rate was 91%, the 50-aug yielded the lowest precision at 57%. This proves that 50-aug was not suitable as a trainable dataset. The YOLOv3 training results point to the amount of LDH cases. The increasing amount of augmented dataset images shows an apparent increase in LDH detection in these four datasets in their specific regions. There were 443 LDH cases separated from L1 to L5 at 20, 62, 112, 144, and 105 (Figure 6A). Our datasets were consistent with the LDH findings from other reports (Faur et al., 2019). The region with the lowest number of LDH was L1-L2 and while the highest symptom occurrence was at L4-L5. L4-L5 was more distinguished in showing the AP value trend than the number of LDH for each lumbar vertebrae region.

From the training results, we can see in each LDH region that the number of symptoms affects deep learning, as shown in Figure 6B. There were only 20 cases of L1-L2, and the AP after training with 50-aug was 23.3%, but with 550-aug AP is the highest at 100%. It shows the limited detection because of deep learning underfitting and overfitting with the small dataset’s insufficient image number. These results were shown practically by the 50-aug AP at 61.9% and the 550-aug at 86.2% in the L2-L3 region with 60 cases of LDH.

This study contributes to the minimum size dataset method to indicate the possibility of training YOLO or other deep learning models. The mAP of the 550-aug group was the highest at 92.4%. However, it shows 550-aug and 350-aug group results through deep learning at each lumbar region. The F1 score was the same at 89%, but the Dice coefficient was 89.4% in the 550-aug group. It displayed the possibility to train the small-scale datasets via the YOLO algorithm.

The LDH case’s uneven distribution indicates that insufficient data volume affects the training and analysis of small-scale datasets. Therefore, data augmentation is one solution to train the deep learning model (Safdar et al., 2020). This study utilizes three methods to augment the datasets, image rotation, contrast, and image exposure to train the algorithm (Figure 5). The MRI image is set to 8-bit grayscale JPEG format. It can control the brightness and darkness to show or hide the image details (Shorten and Khoshgoftaar, 2019). The LDH MRI shows more information and edges such as cauda equina, dural sac, and intervertebral disc from the sagittal view. Except for the 50-aug dataset in the L1-L2 region, this study uses data enhancement processing techniques to diagram the training results. This displayed the trends towards smoother AP in every lumbar region with all of the datasets.

This study compares to other related studies to evaluate the LDH MRI detection performance based on the lumbar spine range (Table 4). Even though, they use different methods and frameworks to research lumbar-related problems, such as Cai et al. (2016) using the converted deep convolutional network (TDCN) and Zhou et al. (2019) with the Siam network to identify and name the lumbar images, Varçın et al.’s (2021) research has better performance for lumbar image recognition regarding precision, accuracy, sensitivity, specificity using YOLOv3.

**TABLE 4:** The comparison of spinal detection performance (%) with the related literature study.

References	Study case	Method	Precision	Accuracy	Sensitivity
Cai et al. (2016)	Lumbar MRI + CT	TDCN + SVM	–	98.1	93.8
Zhou et al. (2019)	Lumbar MRI	Siamese network	98.9	98.6	98.9
This study (550-aug)	Lumbar MRI	YOLOv3	87.0	81.1	91.7

Note: MRI, magnetic resonance imaging; CT, computed tomography.

This deep learning model applies the YOLOv3 algorithm to detect lumbar location using the data augmentation with a small-scale lumbar MRI dataset. It has been proven that this method acquires an effective object detection model with YOLOv3. Compared with other studies, Cai et al. (2016) used 60 volumes of MRI, and 90 volumes of CT to 2D scanning images, and Zhou et al. (2019) used 2,739 images to train for deep learning. They used more than a thousand images in the dataset. This study used only 550 images of lumbar MRI to train YOLOv3. These results show the competitiveness of this study with the other studies in the limited amounts of images.

There are two limitations to this study. The first limitation is using a specific version of the YOLO model but not the latest algorithm. As time progressed YOLO v4 and v5 utilize several new features. YOLOv3 still presents a mature method of object detection for medical image detection (Safdar et al., 2020; Ünver & Ayan, 2019; Varçın et al., 2021). Additionally, the latest YOLO model forked into two new versions named v5s and v5m with different concepts to train, validate, and test the YAML files and additional new augmentation skills to improve performance (Malta, Mendes, and Farinha, 2021).

Moreover, the second limitation is that the definition of this study detects the sagittal view regions of LDH. Although the other report uses U-net architecture to classified lumbar axial view MRI (Mbarki et al., 2020). We used the object detection method with the dataset of sagittal view LDH images as a preliminary study. Despite LDH covering the most common clinic cases of lumbar disc bulging, protrusion, and extrusion (Y. J. Hung et al., 2014), this study focuses on the detection of LDH regions rather than the prediction of symptoms with YOLOv3. Some reports showed an association among disc morphology, disc bulging, and protrusion at the L3-L4, L4-L5, and L5-S1 levels (Breton, 1991; I. Y. Hung et al., 2021). Many asymptomatic people over 40 years old show disc bulging and protrusion (Brinjikji et al., 2015). The introduction of severe LDH symptoms was insufficient to support deep learning in this study. The subjects were from market labor and general outpatients, presenting primarily lumbar bulging and protruding, to a lesser degree extrusion and sequestration.

Therefore, YOLOv3 trains the object detection model with the lumbar disc regions from L1 to L5 associated with disc morphology features at the lumbar disc. It could be the reason for the results of high mAP and AP in this section. This result shows the future utility of this technology as well as possibilities to affect clinical workflow. From our point of view, this form of deep learning detection does not aim to replace any medical personnel but provide support in clinical decision-making through giving quickly accessible, additional information to speed initial investigation, and helping to focus on areas of interest. This will increase diagnosis speed and certainty before the team interdisciplinarily arrives at a decision regarding future operation or treatment (Yang et al., 2021).

For further study, we will reinforce the research team and recruit surgeons specialized in spinal pathology and anatomy. We warrant the performance of the different versions of YOLO object detection models to select the most suitable for fine-tuning on the regions of the LDH dataset. Furthermore, we would explore more cases with various LDH characteristics, such as the height of vertebrae discs alignment and the curvature of the spine, and even use lumbar axial view MRI, applying numerous and advanced deep learning methods.

## Conclusion

This study proposed a method for deep learning training with small-scale MRI datasets for biomedical images. The data augmentation technique plays a key role in YOLOv3 training and detection results. It presents a high score in deep learning model performance with a small-scale dataset. The best results show the mAP of 92.4%, F1 score of 89%, and Jaccard index of 92.9 by training 550 lumbar vertebrae MRI images. The proposed method presents the ability to detect lumbar disc herniation and predict abnormal locations automatically. This method displays the high possibility of initial rapid tests in real-time and auto-detection of the lesion for the clinical diagnosis. This study employed YOLOv3 to detect small part object problems as LDH MRI with a limited dataset of 50–550 images. The results of this study show good competition ability with other tasks for spinal injuries. It also offers the possibility for real-time detection and clinical assistance applications.

## Data Availability

The datasets presented in this article are not readily available because the data that support the findings of this study are available from the corresponding author upon reasonable request. Requests to access the datasets should be directed to cwlung@asia.edu.tw.

## References

[B1] Abdelhafiz D. Yang C. Ammar R. Nabavi S. (2019). Deep Convolutional Neural Networks for Mammography: Advances, Challenges and Applications. BMC Bioinformatics 20 (Suppl. 11), 281. 10.1186/s12859-019-2823-4 31167642 PMC6551243

[B2] Amin R. M. Andrade N. S. Neuman B. J. (2017). Lumbar Disc Herniation. Curr. Rev. Musculoskelet. Med. 10 (4), 507–516. 10.1007/s12178-017-9441-4 28980275 PMC5685963

[B3] Azimi P. Yazdanian T. Benzel E. C. Aghaei H. N. Azhari S. Sadeghi S. (2020). A Review on the Use of Artificial Intelligence in Spinal Diseases. Asian Spine J. 14 (4), 543–571. 10.31616/asj.2020.0147 32326672 PMC7435304

[B4] Breton G. (1991). Is that a Bulging Disk, a Small Herniation or a Moderate Protrusion?. Can. Assoc. Radiol. J. 42 (5), 318. 1933496

[B5] Brinjikji W. Luetmer P. H. Comstock B. Bresnahan B. W. Chen L. E. Deyo R. A. (2015). Systematic Literature Review of Imaging Features of Spinal Degeneration in Asymptomatic Populations. AJNR Am. J. Neuroradiol 36 (4), 811–816. 10.3174/ajnr.A4173 25430861 PMC4464797

[B6] Cai Y. Landis M. Laidley D. T. Kornecki A. Lum A. Li S. (2016). Multi-modal Vertebrae Recognition Using Transformed Deep Convolution Network. Comput. Med. Imaging Graphics 51, 11–19. 10.1016/j.compmedimag.2016.02.002 27104497

[B7] Dao T. Gu A. Ratner A. J. Smith V. De Sa C. Ré C. (2019). A Kernel Theory of Modern Data Augmentation. Proc. Mach Learn. Res. 97, 1528–1537. Retrieved from Available at: https://www.ncbi.nlm.nih.gov/pmc/articles/PMC6879382/pdf/nihms-1047381.pdf. 31777848 PMC6879382

[B8] Faur C. Patrascu J. M. Haragus H. Anglitoiu B. (2019). Correlation between Multifidus Fatty Atrophy and Lumbar Disc Degeneration in Low Back Pain. BMC Musculoskelet. Disord. 20 (1), 414. 10.1186/s12891-019-2786-7 31488112 PMC6729014

[B9] Forsberg D. Sjöblom E. Sunshine J. L. (2017). Detection and Labeling of Vertebrae in MR Images Using Deep Learning with Clinical Annotations as Training Data. J. Digit Imaging 30 (4), 406–412. 10.1007/s10278-017-9945-x 28083827 PMC5537089

[B10] Hung I. Y.-J. Shih T. T.-F. Chen B.-B. Guo Y. L. (2021). Prediction of Lumbar Disc Bulging and Protrusion by Anthropometric Factors and Disc Morphology. Ijerph 18 (5), 2521. 10.3390/ijerph18052521 33806268 PMC7967385

[B11] Hung Y.-J. Shih T. T.-F. Chen B.-B. Hwang Y.-H. Ma L.-P. Huang W.-C. (2014). The Dose-Response Relationship between Cumulative Lifting Load and Lumbar Disk Degeneration Based on Magnetic Resonance Imaging Findings. Phys. Ther. 94 (11), 1582–1593. 10.2522/ptj.20130095 24970094

[B12] Hussain Z. Gimenez F. Yi D. Rubin D. (2017). Differential Data Augmentation Techniques for Medical Imaging Classification Tasks. AMIA Annu. Symp. Proc. 2017, 979–984. Retrieved from Available at: https://www.ncbi.nlm.nih.gov/pmc/articles/PMC5977656/pdf/2730723.pdf. 29854165 PMC5977656

[B13] Hussain Z. Gimenez F. Yi D. Rubin D. (2017). Differential Data Augmentation Techniques for Medical Imaging Classification Tasks. Paper presented AMIA Annu. Symp. Proc. 16, 979–984. 10.1145/3018896.3066906 PMC5977656 29854165

[B14] Jensen M. C. Brant-Zawadzki M. N. Obuchowski N. Modic M. T. Malkasian D. Ross J. S. (1994). Magnetic Resonance Imaging of the Lumbar Spine in People without Back Pain. N. Engl. J. Med. 331 (2), 69–73. 10.1056/nejm199407143310201 8208267

[B15] Malta A. Mendes M. Farinha T. (2021). Augmented Reality Maintenance Assistant Using YOLOv5. Appl. Sci. 11 (11), 4758. Retrieved from Available at: https://www.mdpi.com/2076-3417/11/11/4758. 10.3390/app11114758

[B16] Martin B. I. Mirza S. K. Spina N. Spiker W. R. Lawrence B. Brodke D. S. (2019). Trends in Lumbar Fusion Procedure Rates and Associated Hospital Costs for Degenerative Spinal Diseases in the United States, 2004 to 2015. Spine (Phila Pa 1976) 44 (5), 369–376. 10.1097/brs.0000000000002822 30074971

[B17] Mbarki W. Bouchouicha M. Frizzi S. Tshibasu F. Farhat L. B. Sayadi M. (2020). Lumbar Spine Discs Classification Based on Deep Convolutional Neural Networks Using Axial View MRI. Interdiscip. Neurosurg. 22, 100837. 10.1016/j.inat.2020.100837

[B18] Ozturk T. Talo M. Yildirim E. A. Baloglu U. B. Yildirim O. Rajendra Acharya U. (2020). Automated Detection of COVID-19 Cases Using Deep Neural Networks with X-ray Images. Comput. Biol. Med. 121, 103792. 10.1016/j.compbiomed.2020.103792 32568675 PMC7187882

[B19] Paolucci T. Attanasi C. Cecchini W. Marazzi A. Capobianco S. Santilli V. (2019). Chronic Low Back Pain and Postural Rehabilitation Exercise: a Literature Review. Jpr 12, 95–107. 10.2147/jpr.S171729 PMC6305160 30588084

[B20] Perez F. Vasconcelos C. Avila S. Valle E. (2018). .Data Augmentation for Skin Lesion Analysis In OR 2.0 Context-Aware Operating Theaters, Computer Assisted Robotic Endoscopy, Clinical Image-Based Procedures, and Skin Image Analysis. Springer, 303–311. 10.1007/978-3-030-01201-4_33

[B21] Redmon J. Divvala S. Girshick R. Farhadi A. (2016). You Only Look once: Unified, Real-Time Object Detection. Paper presented Proc. IEEE Conf. Comput. Vis. pattern recognition. 10.1109/cvpr.2016.91

[B22] Redmon J. Farhadi A. (2018). Yolov3: An Incremental Improvement. arXiv preprint arXiv:1804.02767.

[B23] Sadykova D. Pernebayeva D. Bagheri M. James A. (2019). IN-YOLO: Real-Time Detection of Outdoor High Voltage Insulators Using UAV Imaging. IEEE Trans. Power Deliv.

[B24] Safdar M. Kobaisi S. Zahra F. (2020). A Comparative Analysis of Data Augmentation Approaches for Magnetic Resonance Imaging (MRI) Scan Images of Brain Tumor. Acta Inform. Med. 28 (1), 29–36. 10.5455/aim.2020.28.29-36 32210512 PMC7085309

[B25] Sánchez-Peralta L. F. Picón A. Sánchez-Margallo F. M. Pagador J. B. (2020). Unravelling the Effect of Data Augmentation Transformations in Polyp Segmentation. Int. J. CARS 15 (12), 1975–1988. 10.1007/s11548-020-02262-4 PMC7671995 32989680

[B26] Scheer S. J. Radack K. L. O'Brien D. R. Jr (1996). Randomized Controlled Trials in Industrial Low Back Pain Relating to Return to Work. Part 2. Discogenic Low Back Pain. Arch. Phys. Med. Rehabil. 77 (11), 1189–1197. 10.1016/s0003-9993(96)90147-1 8931535

[B27] Shorten C. Khoshgoftaar T. M. (2019). A Survey on Image Data Augmentation for Deep Learning. J. Big Data 6 (1), 60. 10.1186/s40537-019-0197-0 PMC8287113 34306963

[B28] Tsai J.-Y. Jan Y.-K. Liau B.-Y. Subiakto R. B. R. Lin C.-Y. Hendradi R. (2020). A Convolutional Neural Network Model to Classify the Effects of Vibrations on Biceps Muscles. Paper presented Int. Conf. Appl. Hum. Factors Ergon.

[B29] Ünver H. M. Ayan E. (2019). Skin Lesion Segmentation in Dermoscopic Images with Combination of YOLO and GrabCut Algorithm. Diagnostics 9 (3), 72. 10.3390/diagnostics9030072 PMC6787581 31295856

[B30] Varçın F. Erbay H. Çetin E. Çetin İ. Kültür T. (2021). End-To-End Computerized Diagnosis of Spondylolisthesis Using Only Lumbar X-Rays. J. Digital Imaging 34, 85–95. 10.1007/s10278-020-00402-5 PMC7887126 33432447

[B31] Yang M. Zheng Y. Xie Z. Wang Z. Xiao J. Zhang J. (2021). A Deep Learning Model for Diagnosing Dystrophinopathies on Thigh Muscle MRI Images. BMC Neurol. 21 (1), 13. 10.1186/s12883-020-02036-0 33430797 PMC7798322

[B32] Zhao Z.-Q. Zheng P. Xu S.-t. Wu X. (2019). Object Detection with Deep Learning: A Review. IEEE Trans. Neural Netw. Learn. Syst. 30 (11), 3212–3232. Retrieved from Available at: https://ieeexplore.ieee.org/document/8627998/. 10.1109/tnnls.2018.2876865 30703038

[B33] Zhou Y. Liu Y. Chen Q. Gu G. Sui X. (2019). Automatic Lumbar MRI Detection and Identification Based on Deep Learning. J. Digit Imaging 32 (3), 513–520. 10.1007/s10278-018-0130-7 30338477 PMC6499854

